# Colloidal dispersions of oxide nanoparticles in ionic liquids: elucidating the key parameters†

**DOI:** 10.1039/c9na00564a

**Published:** 2020-01-20

**Authors:** J. C. Riedl, M. A. Akhavan Kazemi, F. Cousin, E. Dubois, S. Fantini, S. Loïs, R. Perzynski, V. Peyre

**Affiliations:** Sorbonne Université, Laboratoire PHENIX 4 place Jussieu, case 51 75005 Paris France emmanuelle.dubois@sorbonne-universite.fr veronique.peyre@sorbonne-universite.fr; Laboratoire Léon Brillouin, UMR 12, CNRS-CEA, CEA-Saclay 91191 Gif-sur-Yvette France; Solvionic SA 195 route d’Espagne, BP1169 31036 Toulouse Cedex 1 France

## Abstract

The combination of ionic liquid and nanoparticle properties is highly appealing for a number of applications. However, thus far there has been limited systematic exploration of colloidal stabilisation in these solvents, which provides an initial direction towards their employment. Here, we present a new and comprehensive study of the key parameters affecting the colloidal stability in dispersions of oxide nanoparticles in ionic liquids. Twelve diverse and representative ionic liquids are used to disperse iron oxide nanoparticles. The liquid interface of these nanoparticles has been carefully tuned in a molecular solvent before transferring into an ionic liquid, without passing through the powder state. Multiscale-characterisation is applied, on both the micro and the nano scale, incorporating both small angle X-ray scattering and dynamic light scattering. The results show the surface charge of the nanoparticles to be a crucial parameter, controlling the layering of the surrounding ionic liquid, and hence producing repulsion allowing efficient counterbalancing of the attractive interactions. For intermediate charges the strength of the repulsion depends on the specific system causing varying levels of aggregation or even none at all. Several samples consist of sufficiently repulsive systems leading to single dispersed nanoparticles, stable in the long term. Thanks to the magnetic properties of the chosen iron oxide nanoparticles, true ferrofluids are produced, appropriate for applications using magnetic fields. The strength and breadth of the observed trends suggests that the key parameters identified here can be generalised to most ionic liquids.

## Abstract 2

Key parameters were identified to obtain long-term stable ionic liquid-based colloids.

## Graphical abstract

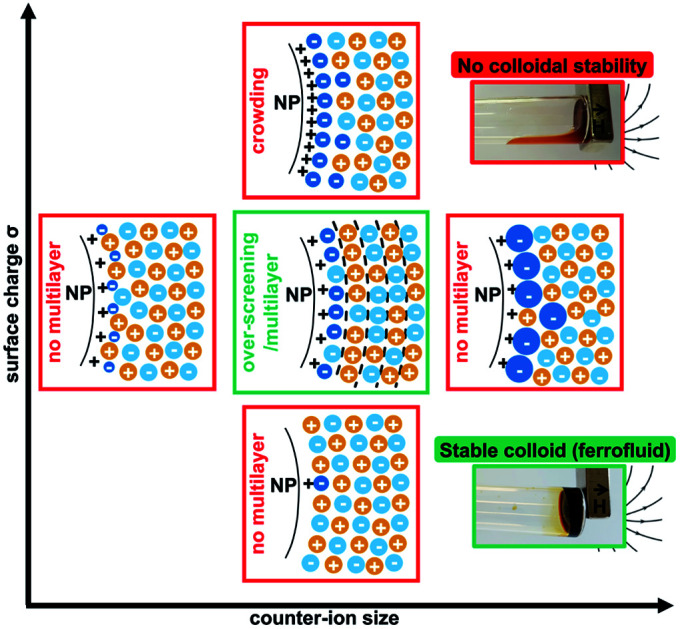

## 1 Introduction

Ionic liquids (ILs) have a variety of properties making them interesting for several fields of applications. For instance, their ability to dissolve many chemical species is used for synthesis and catalytic applications.^1^ Their high thermal stabilities together with their low vapor pressures are useful for seals and bearings.^2^ Their additional modest conductivities and high electrochemical stabilities make them interesting for thermoelectric applications.^3–5^ Adding nanoparticles (NPs) to ionic liquids can further improve the system's properties.^6^ The addition of charged nanoparticles, for example, can improve the thermoelectric properties compared to the use of a solvent alone.^7,8^ Increasing the electrical conductivity of colloidal dispersions provides new possibilities, for example, complex electrolytes for electrochemical devices.^6^

In the past, several approaches were used to stabilise nanoparticles in ionic liquids. One way is grafting of polymers to the surface of nanoparticles. If the ionic liquid is a good solvent for the polymer, the polymer swells in the solvent and causes steric repulsion. Stable colloids of this type were reported in EAN,^9,10^ EMIM X (X = AcO,^9^ EtSO_4_ ^11^), BMIM PF_6_,^12^ and OctylMIM TFSI.^12^ These acronyms and the following are listed in the Abbreviations section.

Another way to achieve stabilisation is based on ion layering of the ionic liquid around the nanoparticles. Molecular dynamics (MD) simulations performed by Zhang *et al.*^13^ were used to calculate the free energy of the interaction between two nanocrystals in a molten salt. Molten salts are the equivalent of ionic liquids based on inorganic species, with a melting temperature above 100 °C. Their simulations showed that the decay length and the amplitude of the force induced by the oscillatory layering of ions are greater than those of van der Waals forces. This leads to colloidal stability. The layering close to the surface was recently experimentally determined by the same group using X-ray pair distribution analysis of nanoparticles in molten salts.^14^ This layering at the interface was used to explain colloidal stability for three types of nanoparticle surfaces: bare (*i.e.* oxides coated with hydroxyl species), coated with small organic ligands, and coated with surfactants having groups that imitate the groups of the ionic liquids. Bare nanoparticles have been reported to form stable colloids in EMIM X (X = AcO,^9^ BF_4_,^15^ SCN,^9^ EtSO_4_ ^13^), BMIM X (X = BF_4_,^13–16^ Cl,^13,14^ Br,^14^ I^13,14^), OHEMIM TFSI,^15^ and DEME TFSI.^15^ However, the surface charge is usually not mentioned. Stable colloids with nanoparticles coated with small organic ligands have been reported in trihexyl(tetradecyl)phosphonium bis(2,4,4-trimethylpentylphosphinate)^13^ and EAN.^17,18^ Surfactants having groups that imitate the groups of the ionic liquids are used to stabilise nanoparticles in EMIM TFSI,^19^ BMIM X (X = TFSI,^20^ BF_4_ ^21^), and C_*r*_MIM X (*r* = 6, 7, 8 and X = TFSI;^2^*r* = 2, 4, 6 and X = BF_4_ ^22^).

However, ionic liquids with their numerous combinations^23^ of anions and cations are very different from molecular solvents and the stability of ionic liquid-based colloidal solutions has been neither perfectly understood nor predictable to date.^24^ Despite the enormous variety of ionic liquids, studies were in general focused on a small number of ionic liquids. This raises the question of whether the results for each system are a specific case and whether these findings are applicable to other systems. Only one group provided an overall picture about colloidal stability, however, mainly in molten salts.^13,14^ Ueno *et al.*^15^ studied the stability of hydrophilic silica particles in 11 ionic liquids, which produced some well dispersed systems according to rheological measurements. The stability was attributed to specific interactions between the surface-bound OH and one of the ions of the ionic liquid.

To the best of our knowledge, no overall experimental study exists on the colloidal stability of nanoparticles in room temperature ionic liquids, with a well described or controlled nanoparticle-ionic liquid interface, yet. Especially in systems with charged nanoparticles, counter-ions are almost never mentioned, although they are essential as they balance the charge in the initial solvent before transferring the nanoparticles to the ionic liquid. However, Mamusa *et al.*^17,18^ and Guibert *et al.*^10^ proved that the nature of these counter-ions influences the colloidal stability in the ionic liquid despite their low concentration compared to the ions from the ionic liquid. In addition, Nordström *et al.* observed an increased stability range of fumed silica particles due to the addition of Li^+^ ions.^25^ These observations are in accordance with studies analysing the nanostructure at the interface between ionic liquids and chargeable surfaces like gold^26^ and graphite.^27^ In all the experiments the interfacial structure was modified when small quantities of Li^+^ or Cl^−^ ions were added and the surface potential was changed.

This work examines routes to properly disperse oxide nanoparticles, in this case (magnetic) iron oxides, in a large variety of ionic liquids, carefully controlling the solid–liquid interface, in order to give an overall picture of stability in ionic liquid-based colloids. The aim is to determine key parameters for colloidal stability, emphasising the role of: (i) the nature of the cations and anions of the ionic liquid, (ii) the nature of the nanoparticles' surface, (iii) the counter-ions, and (iv) the method of transfer of the nanoparticles to the ionic liquid. The characterisation methods of optical microscopy, transmission electron microscopy (TEM), Dynamic Light Scattering (DLS), small angle X-ray/neutron scattering (SAXS/SANS) and magnetic measurements are used in order to determine if these systems are suitable for applications. Moreover, the colloidal dispersion stability is examined over time and under a magnetic field.

## 2 Experiments

### 2.1 Materials

**Fig. 1:**
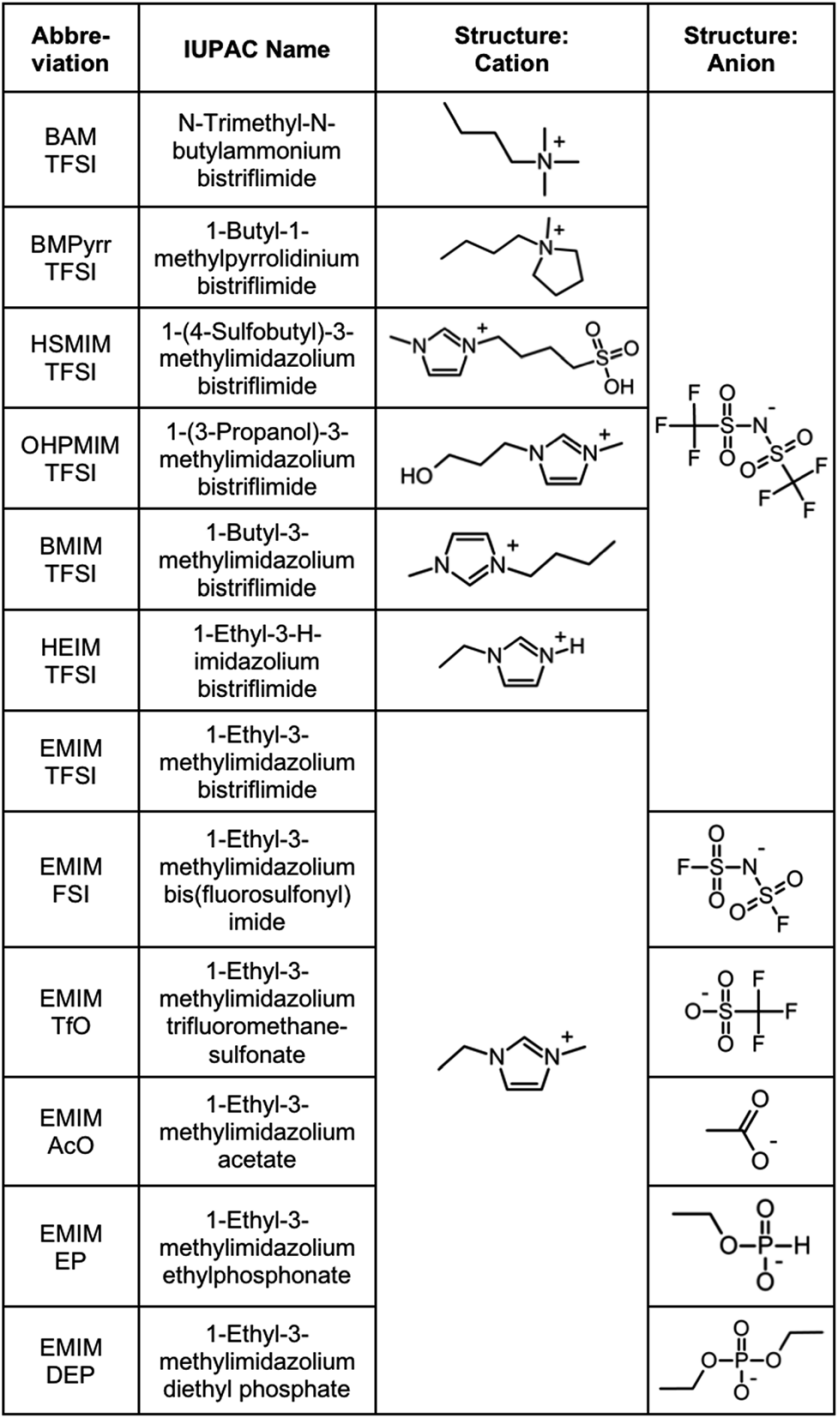
Ionic liquids studied (properties in Table S-1, ESI†).

The products purchased and used as received are listed in Section S-1.1 of the ESI† except for the ionic liquids, which are listed in Fig. 1.

### 2.2 The selected ionic liquids

Ionic liquids are chosen in order to test several families of cations and anions, stable at temperatures equal to or higher than 200 °C, Fig. 1. In one series of seven ionic liquids the cation is varied keeping the anion the same, in this case TFSI. Cations based on imidazolium, pyrrolidinium and ammonium are tested as well as the influence of the substituents on the imidazolium core. In the other series of six ionic liquids the cation was kept the same (EMIM) and the anion was varied (anions including fluorinated, carboxylic and phosphorus groups). Their properties (water content, water uptake, density, viscosity, refractive index, and miscibility with water) are shown in Table S-1 of the ESI.† All ionic liquids were supplied by SOLVIONIC.

### 2.3 Dispersion of nanoparticles in molecular solvents

**Fig. 2:**
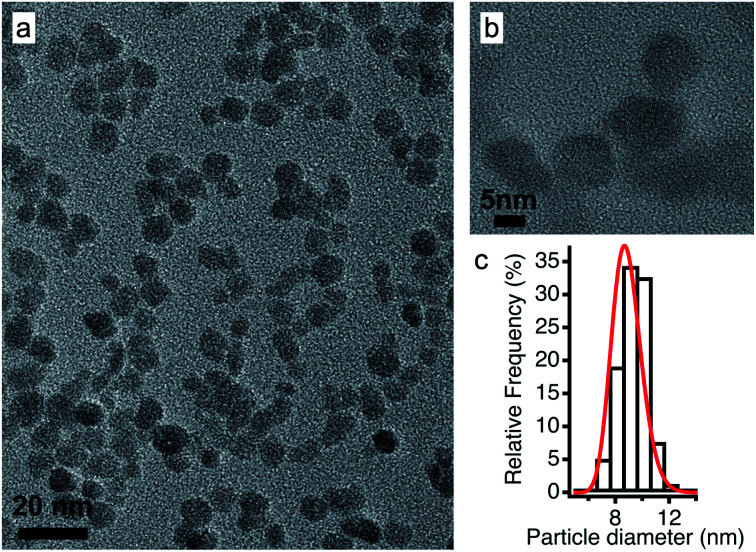
(a) TEM and (b) HRTEM images of the maghemite nanoparticles and (c) their size distribution fitted using a log-normal distribution.

In view of applications, the synthesis of nanoparticles in water is much cheaper and simpler than in ionic liquids. In addition, among oxides, iron oxides are easy to produce as nanometric particles. This is why maghemite iron oxide is chosen as it is formed at room temperature in water. In this work, the same batch of maghemite (γ-Fe_2_O_3_) nanoparticles is used for all experiments. It is prepared by co-precipitation in water of Fe^II^ and Fe^III^ solutions.^28^ The detailed synthesis is described in Section S-1.2 of the ESI.†Fig. 2 shows a TEM image (a) and a HRTEM image (b) of these particles. Their diameter is obtained from a log-normal fit of the size distribution, shown in (c), with a median diameter of 8.7 nm and a polydispersity index *σ* = 0.3.

After the synthesis in water, the pH of the dispersion is around 2 and the nanoparticles bear a positive surface charge compensated by nitrate counter-ions. In the second step, their interface (ligand and counter-ion) is modified (still in water) and they can be transferred to DMSO^29^ in order to elucidate the importance of the initial molecular solvent (water or DMSO). In these molecular solvents, the sign of the charge of the oxide surface can be changed by varying the pH with acids or bases, leading to a positive or a negative surface, respectively. In addition, the nature of the surface charge can be modified. For more details see Section S-1.3 of the ESI.†

Here, three types of nanoparticle surfaces are studied to test the function of the surface: bare nanoparticles with hydroxyl groups on the surface (that can be protonated or deprotonated), particles coated with citrate molecules and nanoparticles coated with a polymer (poly acrylate or poly(acrylate-*co*-maleate)). Besides the sign of the structural charge and the nature of the charged group producing it, a third significant parameter is the nature of the counter-ions of the nanoparticles which compensate the charge. Li^+^, Na^+^, NH_4_^+^, TBA^+^, and BMIM^+^ are used for anionic particles and nitrate (NO_3_^−^), perchlorate (ClO_4_^−^), benzenesulfonate (C_6_H_6_O_3_S^−^), TFSI^−^, and SMIM^+/−^ TFSI^−^ are used for cationic ones. Among these, SMIM^+/−^ TFSI^−^ is the deprotonated form of the ionic liquid HSMIM TFSI. Indeed, the sulfonate group is a strong acid in water, and thus the cation HSMIM^+^ dissociates to form the zwitterion SMIM^+/−^ and the resulting entity SMIM^+/−^ TFSI^−^ is anionic. In water or DMSO, as the structural charge of the nanoparticles is large (around 2 elementary charges per nm^2^), condensation of a large number of the counter-ions on the surface occurs. This defines the effective charge of the moving nanoobjects in the liquid and therefore of the dispersed oxide nanoparticles together with the condensed counter-ions. These condensed ions are crucial as their presence in the moving objects can completely modify the properties of the nanoparticles, for instance their thermodiffusion.^30^ Finally, the amount of free electrolyte can be changed in order to modulate the range of the electrostatic repulsive interaction in molecular solvents. This parameter will not be explored here.

The volume fraction of nanoparticles in the initial molecular solvent *Φ*_NP_ is 1 vol%, except for most of the nanoparticles coated with polymers, for which *Φ*_NP_ = 0.1 vol%.

### 2.4 Routes of transfer towards ionic liquids

**Fig. 3:**
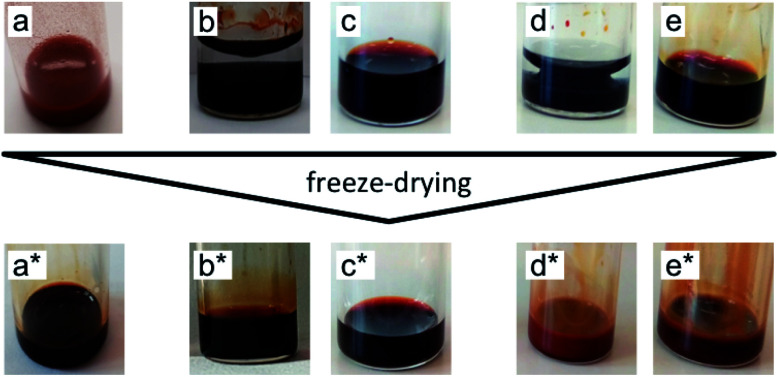
Pictures of the samples before and after freeze-drying for possible transfer routes including at least one stable phase during the process. (a) An unstable sample becomes stable after freeze-drying (a*); stable (b) two-phase and (c) one-phase samples remain stable after freeze-drying (b* and c*); stable (d) two-phase and (e) one-phase samples become unstable after freeze-drying (d* and e*).

In order to avoid any irreversible aggregation, which is likely to occur in the dry state, the particles are kept solvated in water or DMSO all through the nanoparticle surface modifications. The dispersions are mixed with a similar volume of the ionic liquids. DMSO is miscible with all the tested ionic liquids unlike water which is immiscible with some of them (tested in 50 : 50 mixtures, Table S-1, ESI†). In the last step, the molecular solvent is removed by freeze-drying. Ionic liquids do not evaporate in this process due to their low vapor pressure. Fig. 3 shows pictures of the samples before and after freeze-drying for different typical situations. Dispersions in ionic liquids can be obtained from initially flocculated systems in water (brown colour, case a). When two phases exist after addition of the ionic liquid, phase transfer towards the ionic liquid rich phase can occur. This phase is always the bottom one as ionic liquids' densities are higher than the ones of water and DMSO (Table S-1, ESI†). Nanoparticles can disperse in this bottom phase (cases b and d), which may not be the case in the ionic liquid after freeze-drying (case d*). When the initial solvent and the ionic liquid are miscible, the nanoparticles can be dispersed in this mixture (cases c and e). However, this does not preclude the result after freeze-drying (case e*). Indeed, a flocculated initial sample can also be flocculated in the ionic liquid after freeze-drying. In conclusion, the initial state of aggregation in the molecular solvent (they are known to be reversible flocs here) and in the intermediate state in the mixture cannot be used to forecast the final state in the ionic liquid.

### 2.5 Analyses of the sample

The ionic liquid-based colloids are analysed on several spatial length scales and over time. Visual observation is performed on the mm-scale. Maghemite absorbs visible light in the blue and green wavelengths. Unstable suspensions appear turbid and brownish due to visible light scattering (Fig. 3a, d* and e*). Regarding the other samples in Fig. 3, stable dispersions appear dark red saturating to black with increasing nanoparticle volume fractions. If the sample looks stable, optical microscopy is performed in order to detect agglomerates on the micrometre scale. If none are observed, the sample is analysed by DLS. If the apparent diameter extracted from DLS is lower than 100 nm, SAXS and SANS measurements are performed. The viscosity of the final dispersions is difficult to determine accurately as it is highly dependent on the water traces^23^ and on the other additives in the dispersions. Therefore, the diffusion coefficient and the apparent diameter determined by DLS are not always accurate (Section S-1.4.4, ESI†). Thus, small angle scattering (SAS, with neutrons (SANS) or with X-rays (SAXS)) is used to study the nanostructure and DLS is restricted to study the sample ageing (properly kept in a dry atmosphere), as the comparison between signals over time is reliable. Note that SAS is preferred to *in situ* TEM, which could be possible as ionic liquids do not evaporate,^9,12,21,24^ because it averages the whole sample and gives more precise information, especially on interparticle interactions. A laboratory SAXS instrument is used (*λ* = 1.54 Å and *Q*-range 0.004–0.2 Å^−1^). However, the samples strongly absorb X-rays due to the ionic liquid itself and due to the iron atoms. Thus, either the transmission is low when using classical capillaries (around 1 mm thick) or the thickness is not accurate when reducing the thickness under 100 microns with home-made cells. As a consequence, absolute intensity cannot be determined and the high *Q* region (>0.1 Å^−1^) is highly noisy. Therefore, SANS is used to determine the absolute intensity, to explore the high *Q* region and to check that X-rays do not damage the samples. The results prove that these SAXS measurements are reliable in the small *Q* range (Fig. S-1a, ESI†). The form factor *P*(*Q*) of the nanoparticles is determined from several measurements at a low nanoparticle concentration in water and this curve is used for all samples in ionic liquids to determine structural factors *S*(*Q*) using *S*(*Q*) = *I*(*Q*)/(*Φ*_NP_ × contrast × *P*(*Q*)).

The value of *S*(*Q* → 0) therefore compares the studied dispersion to a “reference” dispersion of individual nanoparticles without interparticle interaction. The difference can be due either to interparticle interaction or to a change of the scattering objects (for example formation of small aggregates). Evolution of the size or shape of the nanoparticles after transfer to the IL can be discarded from the scattered intensity at a large *Q*. As normalized intensities in water and the ionic liquid are similar, the nanoparticles are the same (shown by the SANS data in Fig. S-1b, ESI†). If the scattering objects are still individual nanoparticles:1
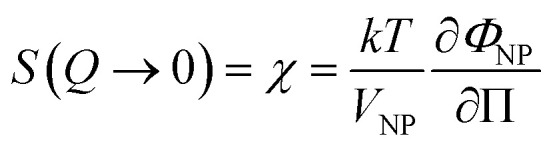
where Π is the osmotic pressure and *V*_NP_ is the volume of the nanoparticle. At a low volume fraction *Φ*_NP_, Π can be developed and *χ* can be derived:2
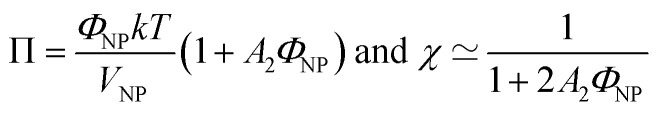


*A*_2_ is the second virial coefficient. For individual nanoparticles, *A*_2_ > 0 corresponds to repulsive interparticle interaction with *χ* < 1, whereas *A*_2_ < 0 corresponds to attractive interparticle interaction with *S*(*Q* → 0) = *χ* > 1. For such individual nanoparticles, it has been shown that stable dispersions of similar maghemite nanoparticles were obtained up to *A*_2_ around −20 which corresponds to *χ* ≃ 1.7 at *Φ*_NP_ = 1 vol% (and *χ* ≃ 1.04 at *Φ*_NP_ = 0.1 vol%).^31^ Higher values of *S*(*Q* → 0) indicate that some aggregation occurs and it measures the number of particles per aggregate.^32^ These aggregates can be stable over time or not, depending on the interactions between them. Hereafter, we will focus on the values of *S*(*Q* → 0) to analyse the differences between the samples.

The colloidal stability of the dispersions is also analysed under a magnetic field, which increases the attractive component of the interparticle interaction and is a good indication of the efficiency of the stabilisation (a field up to 716 kA m^−1^ = 9000 Oe is applied). The iron concentration is determined by flame atomic absorption spectroscopy (FAAS) in order to obtain the nanoparticle volume fraction. The technical details and the implementation of the techniques are detailed in Section S-1.4 of the ESI.†

## 3 Results and discussion

**Fig. 4:**
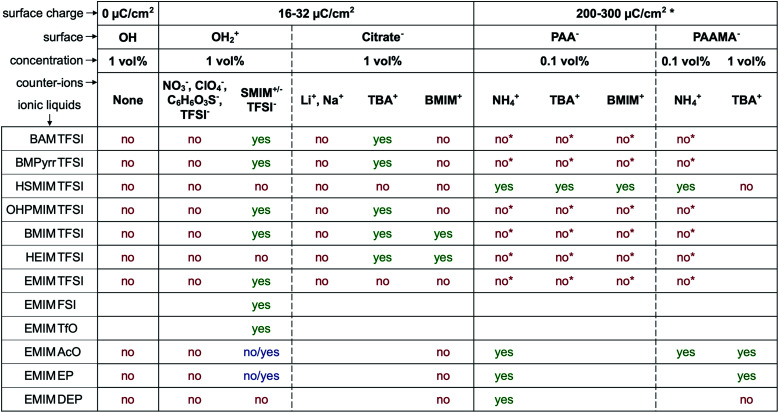
Stability observed by eye and optical microscopy. “Yes” means that at least one route produces dispersions with no aggregates on the micron scale. “No/yes” means that the sample stabilised over time. “No” means that dispersions with aggregates on the micron scale or bigger are obtained. Groups on the surface: OH: neutral hydroxyl ending groups on the nanoparticles at the point of zero charge of the nanoparticles; OH_2_^+^: hydroxyl protonated ending groups on the nanoparticles; citrate^−^: citrate coated nanoparticles; PAA^−^ and PAAMA^−^: polymer coated nanoparticles (poly acrylate and poly(acrylate-*co*-maleate), respectively); SMIM^+/−^ TFSI^−^: butylsulfonate methylimidazolium bistriflimide (SMIM^+/−^ being a zwitterion); NO_3_^−^: nitrate; ClO_4_^−^: perchlorate; C_6_H_6_O_3_S^−^: benzenesulfonate; TFSI^−^: bistriflimide; TBA^+^: tetrabutylammonium; BMIM^+^: butylmethylimidazolium; NH_4_^+^: ammonium.* In the case of a collapsed polyelectrolyte. If the polymer is not collapsed, part of the charge is on the surface of the oxide and part in the solvent, which is a different and more complicated situation. See the text for more information.

The first systematic observation is the impossibility of transferring uncharged nanoparticles whatever the tested ionic liquid (first column in Fig. 4). The nanoparticles thus need to have a charge in the initial molecular solvent, a condition which is necessary but not sufficient. Several other parameters have a decisive impact as shown in Fig. 4. These parameters are the nature of the initial counter-ions, the nature of the nanoparticle surface, the nature of the ionic liquid and the route of transfer (this latter parameter is not shown here but is shown in Table S-2 of the ESI†).

Fig. 4 gives the first assessment of the stability by visual observation using optical microscopy, usually confirmed by DLS. In the second step SAXS measurements of homogeneous samples allow analysis of the nanostructure of the dispersions. The measured scattered intensity is reproducible while performing several measurements on one sample or on different samples prepared in the same way. The *S*(*Q* → 0) values (given in Table S-2, ESI†) range between 0.6 (repulsive interaction) and 4.8 (presence of aggregates of approximately 5 nanoparticles). This means that the scattering objects range from individual particles to small aggregates of a few nanoparticles depending on the exact composition.

### 3.1 Routes of transfer towards ionic liquids

**Fig. 5:**
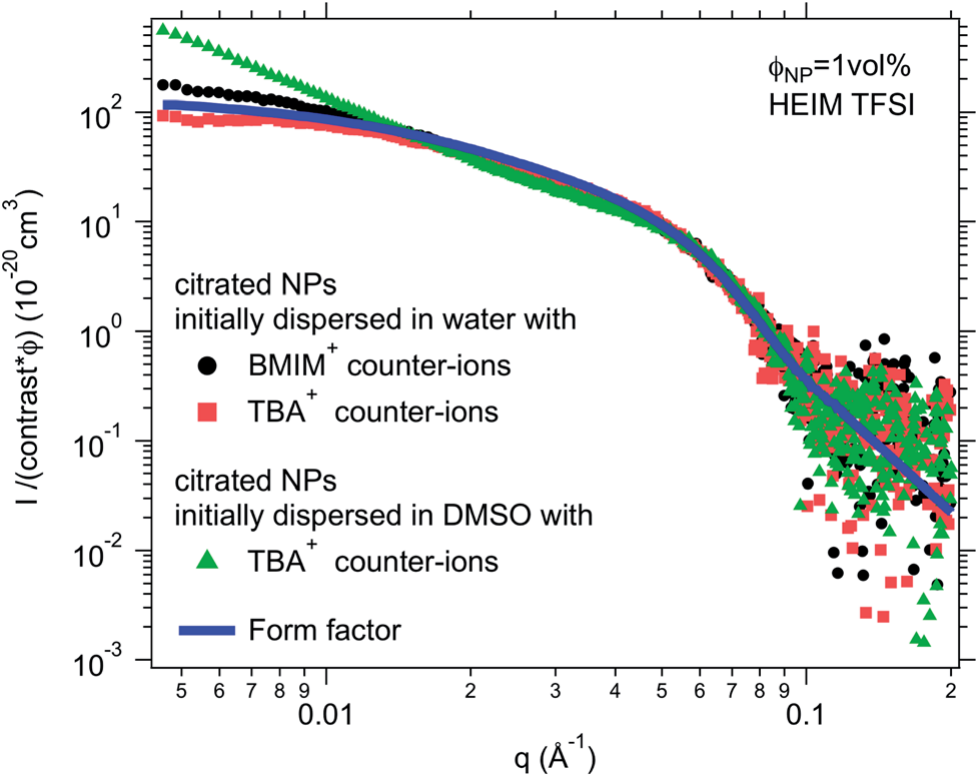
SAXS absolute scattered intensities (normalized by the nanoparticle volume fraction *Φ*_NP_ ≃ 1 vol% and their contrast with the solvent) for dispersions of citrated maghemite nanoparticles in HEIM TFSI with different counter-ions (BMIM^+^ and TBA^+^) and as a comparison with the same counter-ion TBA^+^ for samples once initially dispersed in water and once in DMSO before transfer to the same ionic liquid HEIM TFSI.

The molecular solvent of the initial dispersion is not always miscible with the final ionic liquid. Therefore, two very different routes of transfer exist: (i) in the case of immiscibility, there is a phase transfer of the particles between the molecular solvent and the ionic liquid; (ii) in the case of miscibility, the system passes through an intermediate state where the nanoparticles are in a 50 : 50 solvent/ionic liquid mixture, that is, a concentrated salt solution. Here water and DMSO were used as the initial solvents, since water is not miscible with all ionic liquids (Table S-1, ESI†) but DMSO is. In most cases, there is no visual difference between these two routes for the resulting dispersion in the ionic liquid. The stability observed by microscopy is the same in most cases for both initial molecular solvents (water or DMSO). However, SAXS shows differences on the nanoscale. Fig. 5 shows one example of SAXS measurements for nanoparticles dispersed in HEIM TFSI with the same citrate coating and TBA^+^ counter-ions but initially dispersed in water or in DMSO before the transfer to the same ionic liquid. The normalized intensity shows differences at small wavevectors *q* < 0.05 Å^−1^ indicating differences in the nanostructure. The sample transferred from DMSO scatters more at small *q*. *S*(*Q* → 0) in this case is 5 although *S*(*Q* → 0) = 0.8 for the sample transferred from water. The interaction is thus repulsive in this latter sample although it is attractive with the formation of small aggregates when transferred from DMSO. This difference will be commented on later.

### 3.2 Nature of the counter-ions

The first idea concerning counter-ions is to closely adapt them to the ionic liquid, choosing one ion of the ionic liquid as the counter-ion. Looking closer at the cases, it can be shown that in most cases this doesn't lead to colloidal stability.

Positive nanoparticles with hydroxyl ligands in the initial solvent cannot be dispersed in any ionic liquid shown in Fig. 4 using the counter-ions nitrate, perchlorate, benzenesulfonate or TFSI^−^, even in ionic liquids containing TFSI as the anion. However, if the anionic entity SMIM^+/−^ TFSI^−^ is used as the counter-ion, these particles can be dispersed in more than half of the tested ionic liquids. Moreover, interestingly, they cannot be dispersed in the ionic liquid HSMIM TFSI, which is based on the same species, and they cannot be systematically dispersed in ionic liquids based on imidazolium cations. In contrast, they can be dispersed in ammonium based ionic liquids (BAM TFSI and BMPyrr TFSI). Keeping the same anion, in this case TFSI, the stability mainly depends on the side chains of the cation, while keeping the same cation, in this case EMIM, the stability depends on the nature of the anion. For example, samples based on ionic liquids with anions containing fluorinated groups (TFSI^−^, FSI^−^, and TfO^−^) yield micron-scale stable dispersions which is not systematically true for samples based on ionic liquids with anions containing phosphorus groups (EP^−^ and DEP^−^).

**Fig. 6:**
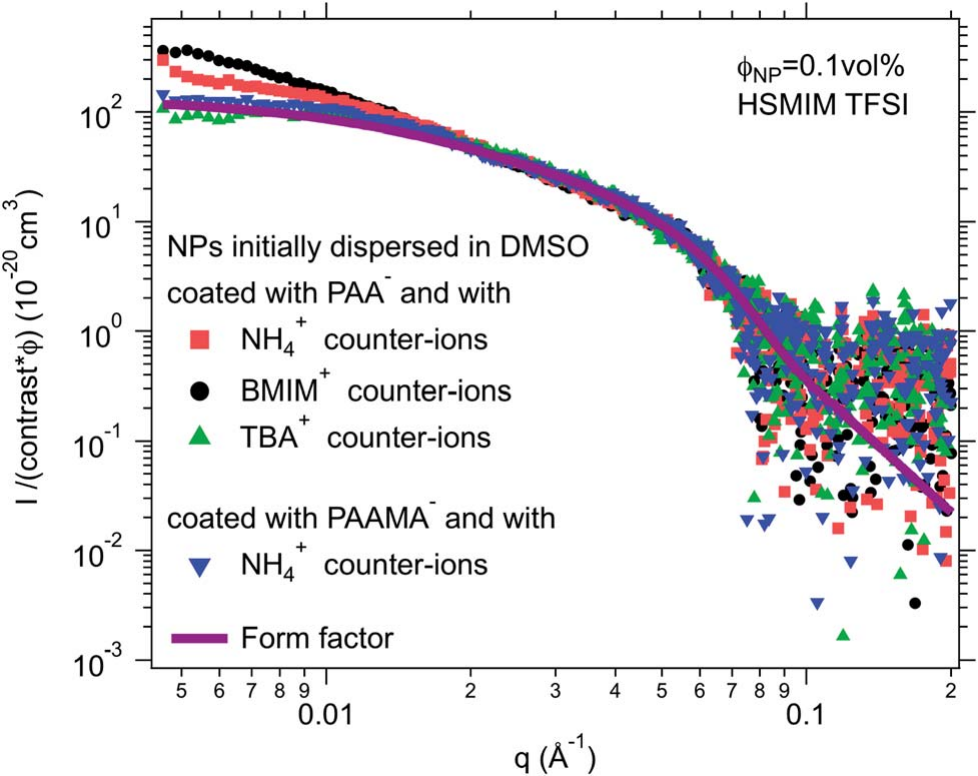
SAXS absolute scattered intensities (normalized by the nanoparticle volume fraction *Φ*_NP_ ≃ 0.1 vol% and their contrast with the solvent) for dispersions of polymer coated maghemite nanoparticles in HSMIM TFSI with different counter-ions for PAA^−^ and as a comparison with the same counter-ion NH_4_^+^ for PAA^−^ and PAAMA^−^ coated nanoparticles.

For negatively charged nanoparticles in the initial solvent (*i.e.* coated with citrate or with a polyelectrolyte), the counter-ions do not play a major role if the charge is a result of short negative polymers based on carboxylic groups. In contrast, their role is crucial if the carboxylic groups come from a small ligand (here citrate). Sodium and lithium initial counter-ions never enable dispersion in contrast to tetrabutyl ammonium (TBA^+^) and BMIM^+^, which lead to nanoparticle dispersions in some ionic liquids. Here again the side groups of the ionic liquid cation (methyl, butyl…) have more influence than the nature of its charged core (imidazolium, pyrrolidinium or ammonium). On the nanoscale however, additional differences between counter-ions are observed that cannot be detected with a microscope. Both TBA^+^ and BMIM^+^ lead to dispersions in HEIM TFSI for similar routes of transfer from water; however, interparticle interactions are weakly repulsive with TBA^+^ and weakly attractive with BMIM^+^ (Fig. 5). For nanoparticles coated with short polymers in HSMIM TFSI, interactions are also weakly repulsive with TBA^+^ while small aggregates are formed with NH_4_^+^ or BMIM^+^ (Fig. 6).

### 3.3 Nanoparticle surface: organisation and charge

Although similar carboxylic groups produce the nanoparticles' charge for polyacrylate-based polymers and small citrate ligands, the nanoparticles cannot be dispersed in the same ionic liquids with these two coatings. Polymer coated nanoparticles are in particular the only ones tested here which can be dispersed in HSMIM TFSI.

In water, these short polymers (around 20 monomers) are strongly adsorbed to the nanoparticles by around ten monomers, the other monomers being free, acting as “hairs”.^33^ Being charged, these “hairs” enable a much higher nanoparticle electric charge to be reached, more than ten times higher than that with citrate ligands. Therefore, these polymers provide stabilisation which is both electrostatic and steric in water.

In the ionic liquid, the charged polymers can still behave as “hairs” around the nanoparticle or collapse on the surface depending on its solubility in the ionic liquid. A steric contribution to the interparticle interaction can exist and/or some contribution resulting from induced organisation due to the charges. This has been seen in EAN where dispersions have been obtained with similar nanoparticles when coated with polyacrylate or with a corresponding polyacrylic uncharged polymer.^10^

Here, the solubility of the polymer PAA^−^ with NH_4_^+^ counter-ions (see Section S-1.3.3 of the ESI† for the experimental details) correlates with the stability reported in Fig. 4 except in HSMIM TFSI. In this latter ionic liquid, the polymer solubilizes after exposure of the ionic liquid to air, which introduces a small percentage of water. As the nanoparticles are originally dispersed in water, some water can remain close to their interface after the transfer to the ionic liquid despite the freeze-drying process, which explains the dispersion of the nanoparticles in HSMIM TFSI despite the insolubility of the polymer in the dry HSMIM TFSI.

In the first situation, *i.e.* the polymer is soluble in the ionic liquid (EMIM X with X = AcO, EP, DEP), dispersions can be obtained; however small aggregates are nearly always present (Table S-2, ESI†). The steric contribution to repulsion brought about by the polymer is thus lower than the highest repulsion that can be obtained without polymers, which is not intuitive. An influence of the nature of the counter-ions nevertheless still exists.

In the second situation, *i.e.* the polymer is not soluble in the ionic liquid, the polymer is collapsed on the nanoparticle, and thus a much smaller steric contribution to repulsion is expected. Despite a large apparent charge, the nanoparticles are not dispersed whatever the nature of the counter-ions. This can originate from a rougher surface or from the higher density of charge due to the polymer (around 32 μC cm^−2^ for citrate and 400 μC cm^−2^ for polyacrylate^33^). This high value can influence the structuring of the ionic liquid at the surface^34–36^ and could be the origin for the difference in colloidal stability between nanoparticles coated with small citrate ligands or larger polymers containing the same carboxylate groups. This will be discussed in more detail later.

### 3.4 Discussion: which are the key parameters?

The presented results confirm the major role of the solid–liquid interface, through the charge of the nanoparticles and the nature of the molecules close to the surface, in order to produce a repulsive contribution to the interaction able to counterbalance the attractive van der Waals (plus in this case also magnetic dipolar) interaction leading to a stable colloidal dispersion in ionic liquids. This generalizes the previous findings in one particular ionic liquid, EAN.^18^ The role of the transfer route and of the nature of the charges is also highlighted here. Moreover, the experiments evidence that, whatever the ionic liquid, a combination can be found in order to obtain colloidal dispersions. What is hidden behind these observations?

Layering of ionic liquids on flat surfaces has been evidenced for a long time using several experimental techniques^37–39^ and numerical simulations.^36,40–43^ It depends on the nature of the surface, on its charge, on the amount of water and on the ionic liquid. This layering is assumed to be at the origin of the colloidal stability in ionic liquids if the number of ionic layers is sufficient. Very recently evidenced on nanoparticles in molten salts, which are cousins of ionic liquids melting at higher temperature, and in BMIM I, the organisation of the ions close to the solid has been studied by X-ray PDF analysis.^14^ This is supported by previous^13^ and new^14^ numerical simulations. These authors correlate the colloidal stability with the affinity of the molten salt for the nanoparticle surface. Their nanoparticles are based on various materials and dispersed in the molten salts after removing the initial ligands present in the original organic solvent used for synthesis, without an additional control of the nanoparticle surface. Here in contrast, we focus on nanoparticles all made from the same material with fine tuning of their surface.

Here the nanoparticle surface is modified in the initial step of the preparation in the molecular solvent, where nanoparticles are charged, this charge being compensated in their neighbourhood by an equal number of counter-ions. The charge on the iron oxide nanoparticles is the structural charge *Z*_NP_. As it is large here (around 1–2 charges per nm^2^, *Z*_NP_ around 250–500 for bare and citrate coated nanoparticles and even more for the polymer coated ones‡‡If the polymer is not collapsed, part of the charge is on the surface of the oxide and part in the solvent, which is a different and more complicated situation. See the text for more information.), a large portion of the counter-ions is condensed on the nanoparticles, resulting in a smaller effective charge at the surface of this layer of bound counter-ions. This effective charge in the molecular solvent is compensated by the free counter-ions which are around the nanoparticles in the diffuse layer. An electrolyte composed of the counter-ions and an ion of opposite charge, *i.e.* a co-ion, is present in the initial colloidal dispersions in molecular solvents, with concentrations usually lower than 0.1 mol L^−1^.

While transferring these nano-objects from the initial molecular solvent towards the ionic liquid, the electrolyte mentioned above is diluted in a much larger concentration of ions of the ionic liquid. Concerning the counter-ions of the nanoparticles, many situations can occur: the initial condensed counter-ions of the nanoparticles can stay around the nanoparticles or they can be exchanged with ions from the ionic liquid. Any intermediate situation can also be encountered. Therefore, the effective charge at the surface of the first layer of bound counter-ions may differ in the ionic liquid compared to the initial value in the molecular solvent.

Considering the spatial distribution of ions, on a random basis, the initial free counter-ions of the nanoparticles should be distributed over the whole volume, and the initial condensed counter-ions could be replaced by the ions of the same charge from the ionic liquid. However, it was observed in EAN that some of the condensed initial counter-ions of sodium and lithium stayed close to the nanoparticle, explaining the difference in the nanostructure in the corresponding dispersions in EAN.^18^ In the present experiments, the influence of counter-ions (Fig. 4–6) also proves that at least some of them stay close to the interface. They generate a particular ionic liquid organisation, and therefore modify the interparticle interactions, the nanostructure and the stability. This influence of the counter-ions can be due to their affinity with cations, anions and the nanoparticles' surface, to their interaction with water, and to their size compared to that of the cations and anions of the ionic liquid. For example, Na^+^ and Li^+^ are small compared to the cations and anions of the scanned ionic liquids, which can strongly influence (destroy or improve) any spatially oscillating structural organisation of ionic liquid ions around the nanoparticles. Similar localisations of species at the interface can be deduced from the experiments of Zhang *et al.*: they managed to disperse nanoparticles in an ionic liquid where these nanoparticles were flocculated by adding a small amount of another ionic liquid which allowed their dispersion.^13^ Localisation of lithium ions added as LiBF_4_ in BMIMBF_4_ on fumed silica particles has also been reported from spectroscopic measurements.^25^ Finally, experiments analysing the nanostructure of ionic liquids at the surface of chargeable flat surfaces like gold^26^ and graphite^27^ show that the interfacial structure is modified when small quantities of Li^+^ or Cl^−^ ions are added and if the surface potential and the resulting density of charge are changed. The influence of the initial counter-ions thus comes from their possible localisation at the solid–liquid interface, which also depends on the nanoparticles' surface charge and on the details of the different molecules at the interface.

It is not only the counter-ions that are important but also the value of the nanoparticles' structural charge *Z*_NP_ on their surface, through its density *σ*. Indeed, the surface structures vary with the superficial surface charge densities *σ*, as evidenced by molecular dynamics simulations^36,40,42,43^ and experiments, for example, on flat conductive surfaces^38,43,44^ changing the electric potential applied on the solid electrode. Looking deeper into the details of the phenomena obtained from simulations,^42^ the structure close to a flat charged surface strongly depends on the surface charge density *σ* and on the nature of the ion of opposite charge in the IL. For this counter-ion, they define *Θ*^max^_ion_, the maximal density of the counter-ions that can be accumulated in the first layer of pure counter-ions close to the charged solid surface. Values of *Θ*^max^_ion_ were calculated by means of molecular dynamics simulations^45^ for several representative ions, not considering the influence of co-ions (the same sign of charge as the surface). The values for the ones used here are ∼45 μC cm^−2^ for BMIM^+^, ∼30 μC cm^−2^ for TBA^+^, and ∼−50 μC cm^−2^ for TFSI^−^. |*Θ*^max^_ion_| is bigger for smaller ions as more ions can fit in the same area. For example, the value for BF_4_^−^ is ∼−100 μC cm^−2^ and such high absolute values are expected here for the alkaline cations sodium and lithium and for the anions nitrate and perchlorate.

On an uncharged surface, where *σ*/*Θ*^max^_ion_ = 0, the ionic liquid is seen to organise in the plane of the surface but not perpendicular to it. The first layer close to the surface contains cations and anions. An increase in the charge, while |*σ*/*Θ*^max^_ion_| < 1, can be compensated by counter-ions in the first layer. Each counter-ion occupies less space than the area per surface charge available. Therefore, the amount of counter-ions in the first layer can be larger than the surface charge. This organisation is sometimes called overscreening in the literature.^46^ If the first layer is not fully filled with counter-ions, then co-ions will complete the layer.^27^ In the direction perpendicular to the surface, layers are formed with an oscillatory/multilayer structure of counter- and co-ions and with a maximal number of layers around |*σ*/*Θ*^max^_ion_| = 0.5. If |*σ*| = |*Θ*^max^_ion_|, a monolayer of counter-ions is formed, which compensates for the surface charge. The ionic liquid structure after this first layer is bulk-like. If |*σ*/*Θ*^max^_ion_|>1, crowding (sometimes called a monotonic structure) occurs, first described by Bazant *et al.*^46^ This means that counter-ions accumulate in more than one layer close to the charged surface because this charge is very high and because the bulky counter-ions cannot compensate for this charge with one dense layer of pure counter-ions. Crowding was observed experimentally by an X-ray reflectivity study^47^ in an ionic liquid containing bistriflimide (TFSI) starting from a surface charge density of ∼50 μC cm^−2^ which is in agreement with the simulated value for *Θ*^max^_ion_.

**Fig. 7:**
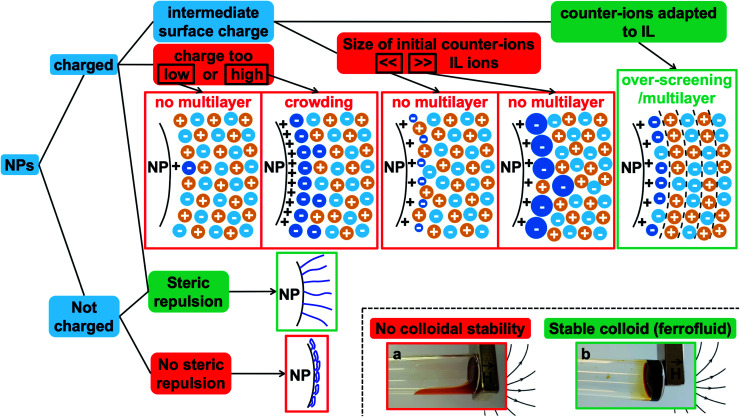
Summary of the different possibilities at the interface of the nanoparticles, charged or not, in an ionic liquid. This indicates the main parameters to tune in order to obtain stable ionic liquid-based colloids. Red boxes: conditions leading to unstable systems. Green boxes: conditions leading to stable dispersions. The diagrams show possible organisations close to the charged solid interface in the case of a positive structural charge of the nanoparticle: the dark blue ions are the initial counter-ions of the nanoparticles, the light blue ions are the counter-ions from the ionic liquid, and the yellow ions are the co-ions of the ionic liquid. The photos show the two situations: on the right of the tube, the magnet traps the liquid when a stable colloidal suspension is obtained as the nanoparticles move with the liquid (b). In contrast, the magnet only traps the nanoparticles when colloidal stability is not reached and the solvent flows at the bottom of the tube (a).

On the basis of all these elements, some of the different possibilities of organisation close to the surface are shown in Fig. 7 in the case of ideal nanoparticles, showing in particular overscreening leading to a multilayer structure or crowding. Additional counter-ions different from the ones of the ionic liquid are considered as they are present in our experiments (they are the initial counter-ions of the particles, localised close to the nanoparticle surface in the ionic liquid). This means that an effective *Θ*^max^_ion_ for a mixed layer of two kinds of counter-ions needs to be defined, which can hugely modify the layering close to the solid compared to a layer with one type of counter-ion. Real nanoparticles are rougher, which will influence the structuring of the ionic liquids compared to a flat surface.^34–36^ Also, the curved surface can influence the layering; however |*σ*/*Θ*^max^_ion_| should remain an important parameter.

Fig. 7 also summarises what these observations tell us about the colloidal stability observed in this study. Firstly, if the nanoparticle has no structural charge prior to the transfer to the ionic liquid, no dispersion will form in any ionic liquid, as we observe here. Indeed, there is no impetus from which an ordered multilayer structure can arise. When this structure is not present there is no phenomenon that can produce any repulsive effect between nanoparticles, and therefore the dispersed nanoparticles are observed to coagulate and sediment.

Secondly, when the surface charge density of the nanoparticle is in the range 16 to 32 μC cm^−2^ (hydroxyl or citrate coated nanoparticles), |*σ*/*Θ*^max^_ion_| remains between 0 and 1 producing layering of the ionic liquid influenced by the nature of the counter-ion. Looking for an example in the case of citrate coated nanoparticles in BMIM TFSI (Fig. 4), both TBA^+^ and BMIM^+^ initial counter-ions facilitate dispersion of the nanoparticles. Differences between the two samples nevertheless appear in the analysis of interactions (Table S-2, ESI†). However, if the initial counter-ions are Li^+^ or Na^+^, which stay at the interface in the ionic liquid, the effective |*σ*/*Θ*^max^_ion_| is lower due to their small size and the structuring induced is not sufficient, which is consistent with the impossibility of stabilising the nanoparticles. Li^+^ or Na^+^ always destroys the stability here (Fig. 4). However, the use of Li^+^ or Na^+^ as initial counter-ions can lead to colloidal stability in the ionic liquid EAN^17,18^ whose cation and anion sizes are smaller than those of the ones tested here. Therefore, the *σ*/*Θ*^max^_ion_ ratio is closer to the optimum of 0.5 and a multilayer structure can be formed that leads to colloidal stability. Moreover, Na^+^ led to better colloidal stability than Li^+^ and EA^+^ as the size matching with the EAN ions is better and therefore the *σ*/*Θ*^max^_ion_ ratio is closer to the optimum of 0.5 for Na^+^. In contrast, the ratio is smaller for Li^+^ and bigger for EA^+^ leading to fewer multilayers and therefore worse/no colloidal stability.

Thirdly, if the charge is much larger (200–300 μC cm^−2^, for collapsed polyelectrolytes), |*σ*/*Θ*^max^_ion_| > 1, which corresponds to the crowding regime. This does not allow the stabilisation of the nanoparticles in the IL, whatever the counter-ion, which confirms that layering of the ionic liquid close to the solid surface is essential.

However, for a given structural charge of the nanoparticles, our results also highlight the influence of both the anions and the cations of the ionic liquid, *i.e.* not only of the counter-ions but also of the co-ions (referring to the charge of the solid surface). Indeed, for example, for positive surfaces obtained with SMIM^+/−^ species condensed on the nanoparticles' surface, taking similar TFSI^−^ ionic liquid anions constituting the layer close to SMIM^+/−^, the dispersion state depends on the nature of the cation (Fig. 4). However, the structural surface charge density does not govern the organization on its own either. Here, for similar *σ* values (positive or negative nanoparticles with citrate), with counter-ions of similar *Θ*^max^_ion_ (TFSI^−^ and BMIM^+^), a dispersion is obtained for negative nanoparticles with BMIM^+^ counter-ions while no dispersion is obtained for positive nanoparticles with TFSI^−^ counter-ions. This is probably due to a variation of *σ*/*Θ*^max^_ion_, which in fact depends on all the species: the sign of the superficial solid charge, nature of this charge, and nature of the co-ions. Indeed, for a given surface, the extent of the layering depends on the cation/anion couple.^35^ The ratio *σ*/*Θ*^max^_ion_ will thus depend on the substituents of the cations (size of the alkyl chains, for example, here HEIM TFSI and EMIM TFSI are not equivalent for dispersing the same nanoparticles) and also on the different interactions (for example, changing the ethyl group for an alcohol as in POMI TFSI, able to form hydrogen bonds). This is why these groups appear more important than the nature of the positive group in the cation. There is also a strong influence of the nature of the anion for a given cation (here EMIM). Indeed, the shape of the anion can modulate the accessibility of the negative charge and thus the interactions with neighbouring species, thus *σ*/*Θ*^max^_ion_. This is consistent with their huge influence on many properties, for example water uptake (Table S-1 in ESI†), conductivities,^48^ or Seebeck coefficients.^49^

Consequently, the strength of the interparticle repulsion is governed not only by the value of the surface charge of the nanoparticles but also by the size and nature of the counter-ions, which either come from the ionic liquids or are different ions introduced on purpose as they can localise at the interface and produce a different organisation of the ions. However, it is not only the ions of opposite sign to that of the surface, *i.e.* the counter-ions, that are important. The co-ions of the same sign are also crucial as the structure results from the coupling of anions and cations.

**Fig. 8:**
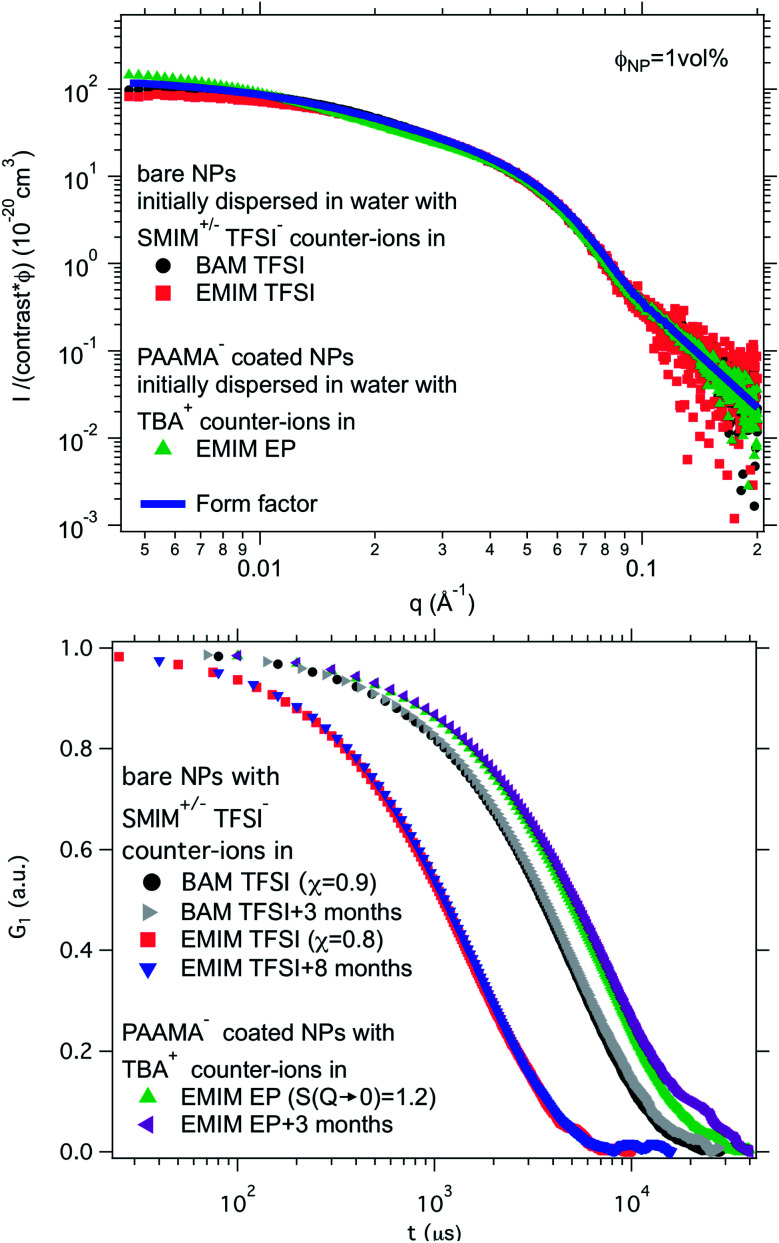
Top: Absolute scattered intensities (normalised by the nanoparticle volume fraction *Φ*_NP_ ≃ 1 vol% and their contrast with the solvent) obtained from SAXS. Bottom: Normalised auto-correlation function *G*_1_ from DLS after preparation and after several months. The shift between the samples is due to their different viscosities at 25 °C. Nanoparticles in BAM TFSI (*η* = 113 cP ^23^) and EMIM TFSI (*η* = 34 cP ^23^) have OH_2_^+^ on their surface and SMIM^+/−^ TFSI^−^ as counter-ions; the nanoparticles in EMIM EP (*η* = 241 cP ^23^) are coated with poly(acrylate-*co*-maleate)^−^ and have TBA^+^ as counter-ions.

Finally, a supplementary parameter, frequently hidden, should be mentioned. Indeed, the remaining water, which is always present in ionic liquids despite careful drying, can hugely modify interfaces if it localises there; therefore it can strongly influence the behaviour. In the present case, water is the initial solvent used for maghemite synthesis and some water can remain after the transfer of the nanoparticles to DMSO and to the ionic liquids. This water content can solvate the oxide surface groups or the counter-ions, if it remains close to the interface. Its influence is moreover very clear looking at the effect of water uptake (by simply keeping the samples in contact with air). It can destabilise some samples in a few seconds (for example all samples based on citrate coated nanoparticles) or can have no impact after several months (for example for bare nanoparticles with SMIM^+/−^ TFSI^−^ counter-ions in EMIM TFSI, the sample shown in Fig. 8a, red squares, and Fig. 8b, red squares and blue triangles).

The role of water in the structure has been directly evidenced from surface force apparatus (SFA) measurements on mica with EMIM TFSI.^39^ The structuring of the ionic liquid close to the mica increased when water was added. This was attributed to a modification of the interface by the solvation of K^+^ counter-ions by water which resulted in an increase of the effective charge of the mica surface. In contrast, for EMIM TFSI on carbon, no difference was detected for the interfacial structure up to water contents of around 3 wt% for surface potentials compatible with AFM measurements.^27^

As a result, no systematic behaviour can be predicted for water as it will depend on its mechanism of action in each system, and therefore on the details of the solid–liquid interface. However, the remaining water or solvent, in this case DMSO, can explain the influence of the route of transfer as the amount and nature of the remaining molecules at the interface can also change the layering and thus the nanostructure. Finally, it should be kept in mind that such remaining molecules of solvent should be considered a tuning parameter as they can either destroy or improve the colloidal stability.

Lastly, recent studies have evidenced the existence of a long-range repulsive force between two mica surfaces of the same charge in ionic liquids.^50–53^ This contribution may also play a role in the colloidal stability in ionic liquids.

## 4 Towards applications

Ionic liquid-based colloids will only be suitable for application if they remain stable during use over a long term. DLS measurements repeated several months apart give information on the long-term evolution of the sample. DLS shows that in all the tested ionic liquids at least one set of parameters leads to a long-term stable sample. It is not only samples with *χ* < 1 (repulsive interparticle interaction) that are stable over months but also samples for which 1 < *S*(*Q* → 0) < 3.5, that is, slightly attractive or with small aggregates. Among these samples stable in the long term, 3 are chosen to illustrate the following situations of interparticle interactions: *S*(*Q* → 0) = *χ* < 1, *S*(*Q* → 0) = *χ* ≃ 1 or *S*(*Q* → 0) > 1 (Fig. 8). At the volume fraction of the experiments, the characteristic time deduced from the DLS correlation curve can be converted to an effective size considering the viscosity of the solvent. However, as already mentioned, the viscosities are not precisely known and the sizes are considered orders of magnitude. The values obtained are nevertheless compatible with the other information on the nanoparticle size from SAXS (Section S-1.4.4, ESI†). Therefore, the shifts shown in Fig. 8 are mainly due to the different viscosities of the solvents. In each solvent, the curves measured months apart overlap for the selected samples, and hence these samples are long-term stable.

The strength of the stability can also be assessed thanks to the magnetic properties of the NPs. An applied magnetic field H induces anisotropic interparticle interaction due to the orientation of the magnetic dipoles of the nanoparticles in the applied field, attractive on average. The maximum value of *H* for which stability is maintained gives an indication of the strength of the repulsive interaction. This is tested by analysing the scattering pattern of a laser beam passing through the sample under the field. The dispersion is stable if no diffraction is observed. If a diffraction line perpendicular to the field direction is obtained,^54^ it means that a second magnetic phase or big aggregates elongated along the applied field are induced. Here several samples are stable up to an applied magnetic field of 716 kA m^−1^ including the three samples shown in Fig. 8. These results confirm that a strong repulsion between particles exists.

**Fig. 9:**
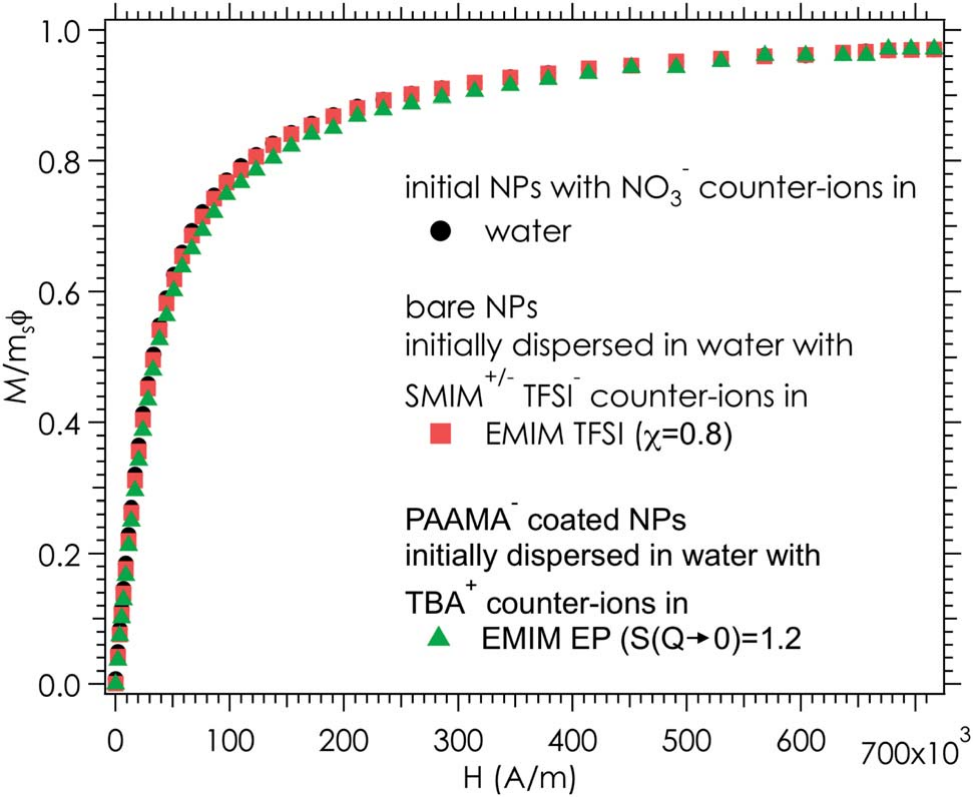
Magnetization divided by the volume fraction of nanoparticles (1.53 ± 0.03 vol% in water, 0.96 ± 0.03 vol% in EMIM TFSI and 1.00 ± 0.03 vol% in EMIM EP) and the saturation magnetization (*m*_s_(water) = 278 ± 17 kA m^−1^, *m*_s_(EMIM TFSI) = 292 ± 18 kA m^−1^ and *m*_s_(EMIM EP) = 278 ± 17 kA m^−1^) as a function of the applied magnetic field *H*. The error bars are around 6%, due to an uncertainty of 0.03% for the concentration determination by FAAS and an uncertainty of 2% for the volume of the ferrofluid in the magnetic measurements.

As the samples are stable under the field, magnetisation curves can be measured. The normalised data are plotted as a function of the applied field (Fig. 9) for two samples in ionic liquids (one case of slightly attractive interparticle interaction and one case of repulsive interaction) and compared with the original sample in water. The similarity of the three curves shows that all NPs are transferred to the ionic liquid, retaining their magnetic properties. Note that this also confirms that the chemical composition of the NPs is stable over time and that their size distribution is maintained.

Consequently, colloidal dispersions stable in the long run have been produced. Due to the magnetic properties of the NPs, they moreover fulfill the definition of magnetic fluids (or ferrofluids).^55,56^ A ferrofluid is a colloidal dispersion of magnetic particles which are magnetic monodomains with a diameter around 10 nm, stable in the long term. It has the usual properties of a liquid but behaves like a liquid paramagnet. It moves as a whole in the direction of the highest magnetic field and retains its liquid properties in a uniform magnetic field *H*. These characteristics make it an interesting liquid for a number of applications, including, for example, sealing, damping and hydrodynamic bearings. Combining this with the properties of ionic liquids leads to the possibility of new applications in high temperature environments, at low pressures and at high voltages, for instance.^2^

## 5 Conclusion

A set of 12 ionic liquids changing either the cation for a given anion or the anion for a given cation is used to disperse iron oxide nanoparticles. In order to identify the key parameters to obtain stable colloidal dispersions, the oxide nanoparticle interface is carefully tuned in a molecular solvent before transferring towards an ionic liquid, without passing through the powder state.

Fig. 7 shows a scheme of the main parameters. Two very different situations occur depending on the charge of the nanoparticles. Uncharged nanoparticles without coatings cannot be directly dispersed in these ionic liquids. However, introducing steric repulsion from adsorbed polymers or other molecules can produce stable colloidal dispersions, a stabilisation method which was not analysed here. In contrast, bare charged nanoparticles can be dispersed thanks to a spatially oscillating multilayer structure formed by the cations and anions of the ionic liquid, which can overcome the attractive contributions to the interparticle interaction. The efficiency of the repulsion depends on the surface occupied by one nanoparticle's counter-ion compared to the area occupied by one superficial charge on the bare nanoparticle. The ratio needs to be between zero and one to create a layering between the solid surface and the bulk ionic liquid. The diagrams in Fig. 7 show that the structural surface charge should not be too low (no layering of the ionic liquid) or too high (crowding of the ionic liquid) in order to induce an appropriate layering of the ions around the nanoparticle.

Another important key parameter is the nature of the nanoparticles' counter-ions initially introduced (they may differ from the ones of the ionic liquid), because of their possible localisation at the solid–liquid charged interface. They modify the layers close to the interface, and thus the dispersion state. As changing the bare charge density of the solid oxide surface is not always experimentally easy, varying these localised counter-ions enables solids and ionic liquids to become compatible.

A coating of charged polymers on the nanoparticles was used to increase the structural surface charge of the nanoparticles and to add a steric contribution to the repulsion. Although stable dispersions can be obtained when the polymer produces a steric repulsive contribution, surprisingly the repulsion is weaker than the one induced by the organisation of ionic liquids on the bare charged nanoparticle surface.

It is not only the nature of the counter-ions that hugely influences the layering at the interface but also the nature of the co-ions and all other species that can locate at the interface like amounts of residual water. This repulsion produced by the layering depends on the geometry and on the nature of the chemical groups of all the species. Therefore, tiny modifications can switch the balance of interaction from repulsive to attractive, and thus switch the structure from individually dispersed nanoparticles to small aggregates.

This study thus identifies the key parameters and suggests that conditions allowing long-term colloidal dispersion can be found in the vast majority of ionic liquids despite their diversity. Indeed, stable colloidal dispersions were produced here whatever the ionic liquid for a particular composition set. However, the exact combination of species which will provide stability for a given ionic liquid cannot be foreseen yet.

Summarising the interesting sample properties for applications, the colloidal dispersions obtained are stable in the long run, and they fulfill the characteristics of ferrofluids, thanks to the magnetic properties of the chosen nanoparticles, retaining their stability under a magnetic field, and they have an increased electrical conductivity compared to the usual molecular solvents, leading to new opportunities.

In the future, it would be interesting to investigate mixtures of ionic liquids and molecular solvents to better understand the role of uncharged molecules. The behaviour at higher temperatures, which is an interesting specific property of ionic liquids, would also be important to explore.

## Conflicts of interest

There are no conflicts of interest to declare.

## Abbreviations

AcO: Acetate
BF_4_: Tetrafluoroborate
BMIM: 1-Butyl-3-methylimidazolium
DEME: *N*,*N*-Diethyl-*N*-methyl-*N*-(2-methoxyethyl)ammonium
C*r* (*r* = 2, 4, 6, 7, 8): Ethyl, butyl, hexyl, heptyl, octyl
EAN: Ethylammonium nitrate
EMIM: 1-Ethyl-3-methylimidazolium
EtSO_4_: Ethyl sulphate
OctylMIM: 1-Octyl-3-methylimidazolium
OHEMIM: 1-(2-Hydroxyethyl)-3-methylimidazolium
PF_6_: Hexafluorophosphate
SCN: Thiocyanate
TFSI: Bis(trifluoromethane)sulfonimide

## Supplementary Material

NA-002-C9NA00564A-s001
