# Supplementary material for: Colloidal dispersions of oxide nanoparticles in ionic liquids: elucidating the key parameters
Source: Nanoscale Adv. 2020 Jan 20;2(4):1560–72. doi: 10.1039/c9na00564a (PMC9419491; doi:10.1039/c9na00564a)
Supplement: NA-002-C9NA00564A-s001 [file NA-002-C9NA00564A-s001.pdf]

Electronic Supplementary Material (ESI) for Nanoscale Advances.
This journal is © The Royal Society of Chemistry 2020

Colloidal dispersions of oxide nanoparticles in ionic liquids: elucidating the key parameters

J. C. Riedl[1], M. A. Akhavan Kazemi[1,], F. Cousin[2], E. Dubois[1,*], S. Fantini[3], S. Loïs[3], R. Perzynski[1], and V. Peyre[1,*]

[1]Sorbonne Université, Laboratoire PHENIX, 4 place Jussieu, case 51, 75005, Paris, France
[2]Laboratoire Léon Brillouin, UMR CNRS 12, CE Saclay, Gif sur Yvette, France
[3]Solvionic SA, 195 route d'Espagne, BP1169, 31036 Toulouse Cedex 1, France.
[*]emmanuelle.dubois@sorbonne-universite.fr, veronique.peyre@sorbonne-universite.fr
[+]these authors contributed equally to this work

SUPPLEMENTARY INFORMATION

S-1 Experimental section

S-1.1 Materials

The following materials were used without further purification:
Citric acid (H_3Cit*H_2O, purity > 99%, NORMAPUR); Poly (acrylic acid sodium salt) (PAA Na, average M_w = 2100 g/mol, SIGMA-ALDRICH); Poly (acrylic acid) (PAA, average M_w = 2000 g/mol, 63wt%, Acros Organics); Poly (Acrylic co-Maleic Acid) solution (PAAMA, average M_w = 3000 g/mol, 50 wt% in H_2O, SIGMA-ALDRICH); ammonia solution (20%, VWR), sodium hydroxide (NaOH pellets, 98%, Acros Organics); dimethyl sulfoxide (DMSO, technical, VWR); Tetrabutylammonium hydroxide 30-hydrate ($TBAOH*30H_2O$, purity > 99%, SIGMA-ALDRICH); $HClO_4$ (65 wt% in water, VWR); hydrochloric acid (HCl, 37% water solution, AnalaR Normapur); iron(II) chloride ($FeCl_2*4H_2O$, AnalaR Normapur, VWR); iron(III) chloride ($FeCl_3*6H_2O$, Prolabo); iron(III) nitrate ($Fe(NO_3)_3*9H_2O$, technical, VWR); acetone (technical, VWR); diethyl ether (purity 99.8%, AnalaR Normapur); Nitric acid (HNO_3, 69.5% water solution, Carlo Erba); Lithium Hydroxide ($LiOH*H_2O$, purity > 99%, Fluka BioChemika); HTFSI (purity 95%, Fluorochem); 1-Methylimidazole ($C_4H_6N_2$, purity 99%, Acros Organics); 1-Bromobutane (C_4H_9Br, purity 99%, Acros Organics).
BMIMOH was prepared from BMIMBr exchanging the anion using an AMBERLITE™ IRN78 resin. The resin was prepared by the following steps: 1) NaOH (1M) was added 2) washing 3) HCl (1M) was added 4) washing 5) NaOH (1M) was added 6) washing; the washing was done with ultra-pure water until the pH was between 5.5 and 7 and the number of moles of base and acid was twice as big as the capacity of the resin. Then, BMIMBr was added and after one hour of stirring the pH was checked and a small quantity was mixed with Ag (NO_3) to test if only BMIMOH was remaining. BMIMBr was prepared by the alkylation of 1-Methylimidazole and 1-Bromobutane following the procedure of reference[1].
Ultra-pure water (Type 1, resistivity of $\simeq$ 18 MΩcm) is produced from potable water by means of a MILLIPORE DIRECT-Q system before use.

S-1.2 Synthesis of magnetic nanoparticles in water

Acidic solutions of $FeCl_2$ and $FeCl_3$ were mixed in a stoichiometric ratio and magnetite (Fe_3O_4) precipitated in a strongly alkaline medium.[5] These spinel type nanoparticles evolve in acidic medium due to their sensitivity to acid attack and oxidation reactions in the presence of oxygen.[6] Therefore, chemical oxidation of magnetite to maghemite at 80°C (γ-Fe_2O_3) was performed with Fe $(NO_3)_3$. This leads to long-term stable (for years) maghemite nanoparticles even in strongly acidic medium. In a final step, after subsequently washing the NPs with acetone and ether, they were dispersed in an aqueous nitric acid solution the pH of which lies between 1.5 and 2. This is called below the "initial ferrofluid". This standard process produces nanoparticles of maghemite, as checked by X-Ray diffraction.[7,8]

S-1.3 Dispersion of the colloidal dispersions in molecular solvent

Several kinds of nanoparticle differing by their surface have been produced in molecular solvents before their transfer to ionic liquids: bare nanoparticles with hydroxyl groups on the surface, citrate coated and polymer-coated nanoparticles. Their

Table S-1. Physical and chemical properties of studied ionic liquids measured at 25°C at PHENIX laboratory*, at SOLVIONIC** or taken from literature. The miscibility of the ionic liquids with water is given for 50:50 mixtures.

short name	CAS	initial water content* in ppm	water uptake in 15min/24h* in wt%	Density in g/cm^3	viscosity in mPa s	refractive index*	miscible with water*
BAM TFSI	25827 3-75-5	563±70	<0.1/0.4	1.41[2]	113[3]	1.409	no
BMPyrr TFSI	223437-11-4	397±50	0.2/0.5	1.39**	81[3]	1.424	no
HSMIM TFSI	909390-59-6	11714±1464	0.1/5.8	1.58*	1711** (22°C)	1.450	yes
OHPMIM TFSI	-	470±59	<0.1/1.3	1.54[2]	109[3]	1.437	no
BMIM TFSI	174899-83-3	573±72	0.1/0.5	1.44[4]	88[3]	1.427	no
HEIM TFSI	-	1231±154	<0.1/1.5	1.57[2]	54[3]	1.423	no
EMIM TFSI	174899-82-2	83±10	<0.1/0.6	1.52[2]	34[3]	1.424	no
EMIM FSI	235789-75-0	48±6	0.1/0.8	1.39[2]	17[3]	1.449	no
EMIM TfO	145022-44-2	324±41	<0.1/3.1	1.38[2]	48[3]	1.434	no
EMIM AcO	143314-17-4	2067±258	0.1/5.1	1.10[2]	127[3]	1.501	yes
EMIM EP	-	72±28	0.2/4.5	1.13[2]	241[3]	1.485	yes
EMIM DEP	848641-69-0	2145±268	0.2/4.6	1.14**	401[3]	1.470	yes

preparation is detailed in the following sections.

S-1.3.1 Bare nanoparticles with hydroxyl groups

1M NaOH aqueous solution was added to 1 vol% of initial ferrofluid in water (prepared as described above, with nitric acid, pH around 1.5) till the point of zero charge (PZC) of maghemite was reached at pH $\approx$ 7. The nanoparticles were magnetically precipitated and the supernatant was removed. The particles were washed several times with ultra-pure water until the free NO_3^- concentration was reduced below 10^{-6} M. At the PZC these uncharged bare nanoparticles have hydroxyl groups on their surface. To test the influence of the nature of the counter-ion, several different aqueous acids were added, including perchloric acid ($HClO_4$), benzenesulfonic acid ($C_6H_5SO_3H$), bistriflimide (HTFSI), and aqueous 1-(4-sulfobutyl)-3-methyl imidazolium bistriflimide (HSMIM TFSI) solution. For each case the pH was reduced to $\approx$ 1.5, leading to stable colloids except for the addition of $SMIM^{+/-}$ $TFSI^-$ solution which led to flocculated nanoparticles in water.

S-1.3.2 Citrate coated particles

The addition of citric acid to 1 vol% of initial ferrofluid in water led to sedimented nanoparticles. They were washed several times with a 10^{-2} M aqueous citric acid solution with the help of magnetic induced sedimentation and removal of the supernatant to obtain a free citric acid concentration of $\approx 10^{-2}$ M. A 1 M base of the type XOH, with X = Li, Na, tetrabutyl ammonium (TBA) or 1-butyl-3-methyl imidazolium (BMIM), was added till a pH $\approx$ 7 was reached, leading to a stable colloid in all cases. The negatively charged citrate coated nanoparticles balance their charge with the cations X of the added base.

S-1.3.3 Polymer coated particles

68% concentrated nitric acid was added to 100 mL of 1 wt% aqueous Poly (Acrylic Acid Sodium salt) solution till a pH of $\approx$ 2 was reached. 50 ml of 1 vol% aqueous initial acid ferrofluid with NO_3^- counter ions was added. After 1 minute of magnetic stirring the nanoparticles were magnetically precipitated and the supernatant was removed. Ammonia solution was added to disperse the nanoparticles at pH $\approx$ 7. In a last step the remaining free ions and polymer is removed by dialysis with 10-12 kDa membranes. The counter-ion is then NH_4. The same steps were applied for Poly (Acrylic co-Maleic Acid) (PAAMA) except the addition of nitric acid in the beginning as the PAAMA solution was already at pH $\approx$ 2. The counter ions were changed by the following steps. First, nitric acid (1M) was added till a pH $\approx$ 2 was reached and the precipitated particles were washed several times. Second, BMIMOH (0.02M) or TBAOH (1M) was added to disperse the nanoparticles at pH $\approx$ 7. In a last step the remaining free ions are removed by dialysis with 10-12 kDa membranes.

The solubility of poly (acrylic acid) was tested in the 12 ionic liquids in order to connect the colloidal stability of PAA^- coated NPs with the solubility of the polymer in the ionic liquids. Therefore, ammonia solution was added to poly (acrylic acid) with 16wt% PAANa and 47wt% PAAH till the ratio of H^+ and NH_4^+ was 1:1 and the pH was close to 7 to exchange H^+ with NH_4^+. NH_4^+ was one of the counter-ions that lead to colloidal stability in some of the ionic liquids. The solution was freeze-dried and heated at 120°C while pumping for 3 hours to remove the water. Around 0.01 mol of the polymer powder per litre of ionic liquid was added. After one day of magnetic stirring the polymer was dissolved in EMIM X (X= AcO, EP, and DEP). The other samples were brought into contact with the moisture of the air and after another week, the polymer was dissolved only in HSMIM TFSI. This can be due to the large water uptake of this ionic liquid, here around 13wt% after 1 week.

S-1.3.4 Transfer from water to DMSO

In contrast to water, dimethyl sulfoxide (DMSO) is miscible with all tested ionic liquids. Therefore, all these different systems were transferred from water to DMSO in order to test the influence of the way of transfer from the molecular solvent towards the ionic liquids. A dialysis step was used, with 10-12 kDa membranes. As the nanoparticles were bigger than the holes in the membrane, only the solvent molecules and the ions were exchanged. The outside DMSO solution with the adapted free ion concentration is changed several times until the water concentration is lower than 10^{-2} vol%. In a last step the different systems were added to the ionic liquid in adapted proportions to obtain, after removing DMSO by freeze-drying, a final colloid with a volume fraction of ≈ 0.01.

Table S-2. Structure factor at low Q, $S(Q\rightarrow 0)$, of ionic liquid-based colloids from SAXS (see text for details). * In the case of collapsed polyelectrolyte. If the polymer is not collapsed, part of the charge is on the surface of the oxide and part in the solvent, which is a different and more complicated situation. See main text for more information. "yes/no" means that the sample destabilised before the SAXS measurements. "no" means that dispersions with aggregates on the micron scale or bigger are obtained and ** means that there are no aggregates on the micron scale but there was not enough sample left to perform the SAXS measurement. OH_2^+: hydroxyl protonated ending groups on the nanoparticles; citrate$^-$: citrate coated nanoparticles; PAA^- and $PAAMA^-$: polymer coated nanoparticles (poly acrylate and poly(acrylate-co-maleate), respectively); $SMIM^{+/-}$ $TFSI^-$: butylsulfonate methylimidazolium bistriflimide ($SMIM^{+/-}$ being a zwitterion); TBA^+: tetrabutylammonium; $BMIM^+$: butylmethylimidazolium; NH_4^+: ammonium.

surface charge →		16-32 µC/cm^2			200-300 µC/cm^2 *				
surface →		OH_2^+	Citrate$^-$		PAA^-			$PAAMA^-$	
concentration →		1 vol%	1 vol%		0.1 vol%			0.1 vol%	1 vol%
counter-ions → / ionic liquids ↓	initial solvent ↓	$SMIM^{+/-}$ $TFSI^-$	TBA^+	$BMIM^+$	NH_4^+	TBA^+	$BMIM^+$	NH_4^+	TBA^+
BAMTFSI	H_2O	0.9	0.8	no	no*		no*	no*	
	DMSO	1.3	1.5		no*	no*	no*	no*	
BMPyrrTFSI	H_2O	2.8	1.4	no	no*		no*	no*	
	DMSO	1.8	1.7		no*	no*	no*	no*	
HSMIMTFSI	H_2O	no	no	no	no		no	2.5	no
	DMSO	no	no		2.6	0.9	3.1	1.1	
OHPMIMTFSI	H_2O	2.0	no	no	no*		no*	no*	
	DMSO	no	1.9		no*	no*	no*	no*	
BMIMTFSI	H_2O	2.8	0.7	yes/no	no*		no*	no*	
	DMSO	1.6	1.7		no*	no*	no*	no*	
HEIMTFSI	H_2O	no	0.8	1.5	no*		no*	no*	
	DMSO	no	4.8		no*	no*	no*	no*	
EMIMTFSI	H_2O	0.7	no	no	no*		no*	no*	
	DMSO	0.9	no		no*	no*	no*	no*	
EMIMFSI	H_2O	1.6							
EMIMTfO	H_2O	1.2							
EMIMAcO	H_2O	yes/no		no	**			2.0	1.8
	DMSO								
EMIMEP	H_2O	1.0		no	1.9				1.2
EMIMDEP	H_2O	no		no	2.6				no
	DMSO								

S-1.4 Techniques

S-1.4.1 Flame Atomic Absorption Spectroscopy (FAAS)

The maghemite-based ferrofluids were dissolved in a concentrated hydrochloric acid solution and their total iron concentration were determined by flame atomic absorption measurements (FAAS) with an Analyst 100 spectrometer from PerkinElmer. The volume fractions ϕ were determined from the total iron concentration in the sample. Taking the molar weight (159.7 g mol^{-1}) and density (5.07 g cm^{-3}) of maghemite,[9] the volume fraction of NPs can be calculated as: ϕ (vol%) = [Fe](mol L^{-1})*1.577.

Table S-3. Magnetisation measurements for the three studied ferrofluids. The error bars for the saturation magnetisation m_s are calculated with an uncertainty of 0.0003 on the volume fraction calculation from the concentration determination by FAAS and 2% for the volume of the ferrofluid in the magnetic measurements.

sample	m_s in kA/m	diameter d_o in nm	polydispersity index σ
NPs with OH_2^+ on their surface and NO_3^- as counter-ions in water	278±17	9.1	0.23
NPs with OH_2^+ on their surface and $SMIM^{+/-}$ $TFSI^-$ as counter-ions in EMIM TFSI	292±18	8.9	0.23
Poly acrylate$^-$ coated NPs with TBA^+ counter-ions in EMIM EP	278±17	9.0	0.25

S-1.4.2 Magnetisation

Room temperature magnetisation measurements were performed on the initial aqueous ferrofluid and two ionic liquid-based ferrofluids using a home-made Vibrating Sample Magnetometer (VSM) up to H=700kA/m. When magnetised, the vibrating sample generates in the measuring coil a voltage at the same frequency proportional to the magnetisation M of the material. The magnetisation is described by the first Langevin's equation, which describes the mean orientation of the magnetic moment under a field H for mono-disperse nanoparticles with a diameter d and a saturation magnetisation m_s. Introducing a lognormal size distribution with a median diameter d_o and a polydispersity σ in the fits, the saturation magnetisation m_s is determined as well as d_o and σ. The characteristics corresponding to the samples presented in figure 6 of the main text are given in table S-3.

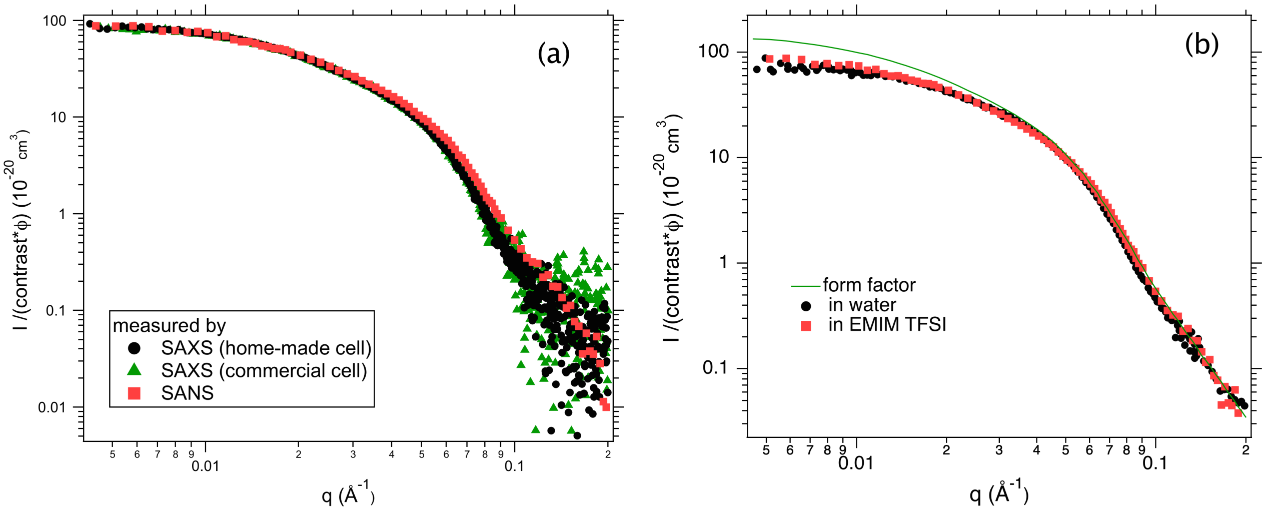

Figure S-1. Absolute scattered intensities (normalized by the NP volume fraction ϕ, here around 1%, and their contrast with the solvent): (a) for dispersions of $SMIM^{+/-}$ TFSI coated maghemite NPs in EMIM TFSI. Two SAXS measurements, once in a home-made cell and once in a classical capillary, are compared to the SANS measurement (this latter providing the absolute level on the Y axis). (b) for the nanoparticles in the initial sample in water and after transfer to the ionic liquid, here as an example the case of EMIM TFSI (same data as (a)), using SANS. The form factor of the nanoparticles is also plotted.

S-1.4.3 Small Angle X-ray and neutron Scattering (SAXS and SANS)

SAXS experiments were carried out with a laboratory XEUSS 2.0 (W)SAXS. The beam energy was fixed at 8 keV and the wavelength at λ=1.54 Å. The sample to detector distance was 3m to yield an accessible Q-range of 0.004 $Å^{-1}$-0.2 $Å^{-1}$. The form factor P(Q) of the NPs is determined from a set of dilute samples at NP concentrations of 0.1, 0.3 and 1 vol% in water (keeping the free electrolyte concentration constant) extrapolating the scattering intensities to the single particle contribution at ϕ=0 vol%. This form factor was used for all the samples in ionic liquids to determine the apparent structure factors S(Q) from the experimental scattered intensity I(Q) using equation 1 from the main paper. The value S(Q$\rightarrow$ 0) is related to the interparticle interactions and the values are given in table S-2. If the particles are individually dispersed, S(Q$\rightarrow$ 0) is the osmotic compressibility χ. A system is attractive if χ>1 and repulsive for χ<1. High values of S(Q$\rightarrow$ 0) indicate the formation of aggregates.
However, as explained in the main text of the paper, the samples strongly absorb X-rays due to the ionic liquids and to iron. Therefore, either the transmission was very low when using classical calibrated polyimide capillaries (thickness 1.5mm) or

the thickness was not accurate when reducing the thickness with home-made cells (thickness < 0.1 μm). As a consequence, absolute intensity cannot be determined and the high Q region (> 0.2 $Å^{-1}$) is highly noisy (see Fig. S-1a).
Therefore SANS were performed on the PAXY spectrometer at the LLB facility (CEA Saclay, France). Three different configurations were used (neutron wavelength λ=6Å, sample to detector distance d=1m; λ=6Å, d=3 m and λ=8.5Å, d=5 m) leading to an accessible Q-range of 0.004 $Å^{-1}$ to 0.2 $Å^{-1}$. Samples were measured in quartz cells with 1 mm inner thickness. Standard correction procedures were performed[10] using the Pasinet software (available free of charge at http://didier.lairez.fr/) in order to determine the absolute intensity (cm^{-1}). The SAXS curves rescaled on the SANS measurement in figure S-1a prove that these SAXS measurements are reliable in the small Q range.
Figure S-1-b shows SANS measurements of colloidal dispersions of the same NPs in water and in an ionic liquid (namely EMIM TFSI). The high Q region where the scattered intensity can be identified to the form factor of the NPs (Q above 0.06 $Å^{-1}$) is properly measured by SANS (by opposition to SAXS in Fig. S-1-a). We then conclude that the nanoparticles keep their shape and their size distribution after transfer from water to the ionic liquid. This conclusion is valid for all dispersions measured by SANS.

S-1.4.4 Dynamic Light Scattering (DLS)

Light scattering measurements were performed using a Vasco DLS Particle Analyzer from Cordouan Technologies to study translational diffusion properties of water-based and IL-based dispersions for volume fractions ϕ ranging from 0.1vol% to 1.53vol%. The laser of the device is operating at a wavelength λ=656 nm. An around 200 μm thin film is analysed in backscattering mode at 135 ° (i.e. Q=2.3*10^{-3} $Å^{-1}$ in water). These conditions prevent both the effects of light absorption by the colloidal suspension and multiple scattering even in strongly absorbing media.[11] Field autocorrelation curves with a precise baseline at long times were obtained by optimizing parameters such as the incident laser power, the sampling time and the number of channels. The measured intensity correlation function G(t) was transformed into $G_1(t)$ using the expression:

$$G_1(t) = [G(t)]^{1/2} - offset \tag{1}$$

The normalized intensity auto correlation functions $G_1(t)$ were analysed with a stretched exponential function, $e^{-(t/\tau)^{\beta}}$ with a distribution of relaxation times described by a decay time τ and a stretching exponent β. The nanoparticle translation time τ is probed. It corresponds to the translational diffusion coefficient $D_t = (<\tau> Q^2)^{-1}$, which, in non-interacting conditions, is related to the hydrodynamic radius R_H using Stokes-Einstein's equation:

$$D_t = \frac{k_B T}{6\pi\eta R_H} \tag{2}$$

where k_B is the Boltzmann constant, T the absolute temperature, η the solvent shear-viscosity (0.89*10^{-3} Pa s for water; see η in table S-1) for ionic liquids; the values are given for T=25°C at which all experiments were performed.
The values for the determined hydro-dynamical diameter d_H of the samples depicted in figure 7b) of the main paper are given in table S-4. The reference hydro-dynamical diameter d_H of the initial sample in water is 13.4 nm for 1 vol% NP concentration. The stretching exponent β is close to 1 for all measurements meaning that there exists only one population of particle size. For NPs with hydroxyl protonated groups on their surface and $SMIM^{+/-}$ $TFSI^-$ as counter-ions the zwitterion concentration is around 2%. This could increase the viscosity of the sample whereas the samples viscosity would be decreased by a higher water content. A plausible order of magnitude of an eventual amount of molecular solvent remaining after freeze-drying is the amount of water taken by the ionic liquid alone after 24 hours at air. Assuming that both contributions influence linearly the viscosity, an average hydro-dynamical diameter d_H can be calculated with equation 2 and an error bar is estimated.

Table S-4. Values for DLS results for the three ferrofluids depicted in figure 7b of the main paper. The error bars for the hydro-dynamical diameter d_H are calculated with a linear contribution of the additive's viscosity to the viscosity value of the pure ionic liquid as explained in the text and an uncertainty of 0.0005 for the refractive index. The second measurement was done after 3 months except for EMIMTFSI (8 months).

sample	d_H (1st measure) in nm	d_H (2nd measure) in nm
NPs with OH_2^+ on their surface and $SMIM^{+/-}$ $TFSI^-$ as counter-ions in BAM TFSI	9.8±1.1	10.2±1.2
NPs with OH_2^+ on their surface and $SMIM^{+/-}$ $TFSI^-$ as counter-ions in EMIM TFSI	9.6±3.1	9.6±3.1
Poly $acrylate^-$ coated NPs with TBA^+ counter-ions in EMIM EP	9.1±0.3	9.8±0.3

References

1. Sirieix-Plénet, J., Gaillon, L. & Letellier, P. Behaviour of a binary solvent mixture constituted by an amphiphilic ionic liquid, 1-decyl-3-methylimidazolium bromide and water. *Talanta* **63**, 979–986, DOI: 10.1016/j.talanta.2004.01.001 (2004).
2. Dong, Q. *et al.* ILThermo: A Free-Access Web Database for Thermodynamic Properties of Ionic Liquids †. *J. Chem. Eng. Data* **52**, 1151–1159, DOI: 10.1021/je700171f (2007).
3. Paduszyński, K. & Domańska, U. Viscosity of Ionic Liquids: An Extensive Database and a New Group Contribution Model Based on a Feed-Forward Artificial Neural Network. *J. Chem. Inf. Model.* **54**, 1311–1324, DOI: 10.1021/ci500206u (2014).
4. Currás, M. R., Husson, P., Pádua, A. A. H., Costa Gomes, M. F. & García, J. High-Pressure Densities of 2,2,2-Trifluoroethanol + Ionic Liquid Mixtures Useful for Possible Applications in Absorption Cycles. *Ind. & Eng. Chem. Res.* **53**, 10791–10802, DOI: 10.1021/ie5008568 (2014).
5. Massart, R. Preparation of aqueous magnetic liquids in alkaline and acidic media. *IEEE transactions on magnetics* **17**, 1247–1248 (1981).
6. Jolivet, J.-P. & Tronc, E. Interfacial electron transfer in colloidal spinel iron oxide. Conversion of Fe3o4-Fe2o3 in aqueous medium. *J. Colloid Interface Sci.* **125**, 688–701, DOI: 10.1016/0021-9797(88)90036-7 (1988).
7. Mamusa, M. *et al.* Microstructure of colloidal dispersions in the ionic liquid ethylammonium nitrate: influence of the nature of the nanoparticles' counterion. *J. Physics: Condens. Matter* **26**, 284113, DOI: 10.1088/0953-8984/26/28/284113 (2014).
8. Berkovski, B. (ed.) *Magnetic Fluids and Applications Handbook* (Begell House Inc. Publ. New York, 1996).
9. Schwertmann, U. & Cornell, R. M. *Iron oxides in the laboratory* (John Wiley & Sons, 2008).
10. Brûlet, A., Lairez, D., Lapp, A. & Cotton, J.-P. Improvement of data treatment in small-angle neutron scattering. *J. Appl. Crystallogr.* **40**, 165–177, DOI: 10.1107/S0021889806051442 (2007).
11. Frot, D., Patin, P., Woodley, B. & Pinier, F. Détecteur d'Intensité Lumineuse Diffusée par des Films de Milieux Colloidaux. *Eur. Pat. Off. 0-654-661-A1* (1994).
